# A Small-Molecule Mitofusin 1 Agonist Enhances Islet Survival Under Hypoxic Conditions In Vitro and Improves Transplantation Outcomes

**DOI:** 10.3390/biom15111585

**Published:** 2025-11-11

**Authors:** Yue Wang, Bofeng Yang, Pengkun Song, Zexiang Ji, Di Zhang, Wenxuan Chen, Lei Du, Lei Liu

**Affiliations:** 1 State Key Laboratory of Organ Regeneration and Reconstruction, Institute of Zoology, Chinese Academy of Sciences, Beichen West Rd., Chaoyang, Beijing 100101, China; 2 Beijing Institute for Stem Cell and Regenerative Medicine, Beijing 100101, China; 3 University of Chinese Academy of Sciences, Beijing 100049, China; 4 College of Life Sciences, Nankai University, Tianjin 300071, China

**Keywords:** mitochondrial fusion, hypoxia, oxidative stress, islet

## Abstract

**Background**: Hypoxia-induced oxidative stress compromises the survival and function of transplanted islets, contributing to high rates of islet transplantation failure. **Methods**: This study investigated the small-molecule mitochondrial fusion agonist S89, which specifically activates mitofusin 1 (MFN1). We assessed its protective effects against hypoxia-induced oxidative stress and apoptosis in pancreatic β-cells. **Results**: In mouse insulinoma cells (Min6), S89 enhanced cell viability by promoting mitochondrial fusion to inhibit mitochondrial reactive oxygen species (mtROS) overaccumulation (S89 reduced mtROS by approximately 30%) and attenuated mitochondrial lipid peroxidation; furthermore, it suppressed hypoxia-induced apoptosis via downregulation of the BAX/BCL-2 ratio, thus protecting the cells from hypoxia-induced oxidative damage. Notably, S89 significantly potentiated glucose-stimulated insulin secretion (GSIS) in both the Min6 β-cell line and primary mouse islets. Critically, S89 pretreatment enhanced hypoxia resistance in islets and significantly increased graft survival upon transplantation into streptozotocin (STZ)-induced type 1 diabetic (T1D) mice, maintaining prolonged blood glucose homeostasis. **Conclusions**: These findings demonstrate that S89 protects β-cells from hypoxic injury, indicating its efficacy as a therapeutic approach for improving islet transplantation outcomes.

## 1. Introduction

Type 1 diabetes (T1D) is an autoimmune-mediated disorder characterized by the destruction of pancreatic β-cells, leading to insufficient insulin biosynthesis [1]. Pancreatic β-cells, which exclusively synthesize and secrete insulin, are central to T1D pathogenesis due to their role in glucose homeostasis [2]. Insulin regulates carbohydrate, lipid, and protein metabolism by activating the PI3K/Akt pathway to promote GLUT4 translocation. This suppresses gluconeogenesis and stimulates glycogen synthesis, lipogenesis, and protein synthesis, thus making it indispensable for maintaining glucose homeostasis and energy balance [3,4]. In patients with type 1 diabetes (T1D), insulin deficiency impairs cellular glucose utilization, forcing a metabolic shift toward free fatty acids derived from triglyceride lipolysis as the primary energy source [5]. In the liver, excessive triglyceride catabolism results in ketone body production; the accumulation of these metabolites lowers blood pH, potentially leading to life-threatening diabetic ketoacidosis [6].

Recently, islet transplantation has emerged as a promising strategy to restore glycemic homeostasis in diabetic patients; however, its clinical application remains limited by critical challenges, including donor scarcity, post-transplantation islet stress, and the requirement for chronic immunosuppression [7]. The native pancreas contains a rich vascular network that supplies oxygen and nutrients to islet cells. However, transplanted islets initially depend on oxygen diffusion from the microenvironment to re-establish a vascular network, which takes around 1–3 months to complete [8]. This prolonged revascularization period creates a severely hypoxic niche, which exacerbates mitochondrial dysfunction, oxidative stress, and ultimately leads to cell death [9]. Additionally, transplanted islets exhibit reduced antioxidant capacity, with β-cells being highly vulnerable to oxidative stress due to their intrinsically low antioxidant defenses, thereby decreasing β-cell viability [10,11]. The majority of transplanted human or animal islets undergo early graft loss, highlighting the critical need to improve the quantity and quality of grafts [12,13]. Therefore, strategies to inhibit islet cell stress and promote islet survival post-transplant are essential for successful islet transplantation.

Mitochondria serve as the primary energy factories of the cell and perform critical physiological functions, including the maintenance of cellular redox homeostasis and regulation of metabolic pathways [14]. In transplanted islets, hypoxia-induced stress disrupts mitochondrial integrity, impairing ATP production, amplifying oxidative damage, and compromising cellular viability [15]. Mitochondria are highly dynamic organelles, the functional fitness of which relies on a balance between fission and fusion as processes essential for quality control, damage repair, and adaptation to stress [16]. Targeting mitochondrial dynamics, specifically by enhancing fusion, represents a promising strategy to bolster mitochondrial resilience under hypoxic conditions. Our laboratory previously identified the small-molecule S89, which selectively activates mitofusin 1 (MFN1), as a key mediator of mitochondrial outer membrane fusion. By binding to the helix bundle 2 (HB2) domain of MFN1, S89 alleviates its autoinhibitory state, thereby promoting GTP hydrolysis and membrane fusion [17]. In models of oxidative stress and ischemia–reperfusion injury, S89-treated cells exhibit improved mitochondrial membrane potential, suppressed ROS accumulation, and increased ATP synthesis. MFN1 plays a critical role in maintaining mitochondrial network integrity, metabolic efficiency, and cell survival in pancreatic β-cells. Dysregulation of MFN1-mediated fusion has been linked to mitochondrial dysfunction and impaired insulin secretion under diabetic conditions [18]. Given these protective effects, we aim to investigate whether S89 can mitigate hypoxia-induced damage in transplanted islets, ultimately improving graft survival and function in diabetic therapy.

The mouse insulinoma cell line MIN6 exhibits many characteristics of normal pancreatic β-cells. Therefore, it serves as a crucial in vitro model for studying β-cell function, insulin secretion mechanisms, drug screening, and islet transplantation in vivo. In this study, we demonstrate that S89 specifically promotes mitochondrial fusion and protects Min6 cells from hypoxia-induced damage. S89 treatment suppresses mitochondrial ROS accumulation, attenuates lipid peroxidation, and inhibits apoptosis by downregulating the BAX/BCL-2 ratio. Critically, S89 pretreatment enhances hypoxia resistance in transplanted islets, significantly improving graft survival and glucose homeostasis in diabetic models. These findings position S89 as a potential therapeutic approach to mitigate hypoxic injury in islet transplantation.

## 2. Materials and Methods

### 2.1. Cell Culture

The mouse pancreatic islet cell line (Min6) was cultured in Dulbecco’s Modified Eagle’s Medium (DMEM) (base medium), supplemented with 10% fetal bovine serum (Vivacell, C04001), 1× MEM non-essential amino acid solution (100×) (Gibco, Cat. 11140050), and 0.05 mM β-mercaptoethanol (β-Mer). All cells were maintained in an incubator at 37 °C with 95% air and 5% CO_2_.

The mitochondrial fusion agonist S89 (provided by Professor Xiao-Jiang Hao, Kunming Institute of Botany, Chinese Academy of Sciences) was dissolved in dimethyl sulfoxide (DMSO) to prepare a 1 M stock solution. Min6 cells were treated with 100 nm S89 for 48 h, followed by 24 h of deferoxamine (DFO, 100 nm, MedChemExpress, Cat. HY-B1625) exposure to induce chemical hypoxia. Min6 cells were co-treated with 100 nM S89 for 48 h by exposing them to a hypoxic environment (1% O_2_, 5% CO_2_, 94% N_2_) in a sealed chamber.

### 2.2. Type 1 Diabetes Model

Male C57BL/6J mice (8–10 weeks) were obtained from the Animal Center of the Institute of Zoology, Chinese Academy of Sciences, with at least 5 mice per group. The T1D model was established via five consecutive days of intraperitoneal streptozotocin (STZ, 75 mg/kg; MedChemExpress, HY-13753, Monmouth Junction, NJ, USA) administration, which selectively destroyed pancreatic β-cells. Mice with fasting blood glucose levels ≥11 mmol/L were selected to ensure typical diabetic pathology. The mice were housed in the animal facility at the Institute of Zoology, Chinese Academy of Science, under standard conditions in accordance with the institutional regulations for integrative scientific research. The IACUC protocol number is 1124072200100. All animal experiments described in this study were approved by the Institutional Animal Care and Use Committee.

### 2.3. Cell Viability Assay

Cell viability was evaluated using the CCK-8 assay (Beyotime Biotechnology, C0038, Shanghai, China). Min6 cells were seeded in 96-well plates until reaching 70–80% confluence, followed by medium replacement and the addition of 10 μL of CCK-8 solution per well. After 2 h of incubation, absorbance at 450 nm was measured using a microplate reader, and cell viability was calculated as the percentage of live cells. Triplicate experiments were performed.

### 2.4. Mitochondrial Reactive Oxygen Species (mtROS) Detection

Mitochondrial reactive oxygen species (mtROS) were quantified using the MitoSOX Red Mitochondrial Superoxide Indicator (Thermo Fisher, M36008, Waltham, MA, USA). Cells were collected and washed twice with PBS before incubation with a 15 μM MitoSOX Red working solution for 30 min at 37 °C. Following centrifugation at 1000 rpm for 5 min, cells were washed twice with PBS, resuspended, and analyzed via flow cytometry to assess mitochondrial superoxide (O_2_•^−^) production.

### 2.5. Lipid Peroxidation Detection

Lipid peroxidation in β-cells was evaluated using the BODIPY-C11 fluorescent probe. Cells were collected, washed twice with PBS, and incubated with 10 μm BODIPY-C11 at 37 °C for 30 min. After centrifugation and washing, cells were resuspended in PBS for flow cytometry analysis. The oxidation of BODIPY-C11 shifts fluorescence from green (500–530 nm) to red (580–610 nm), reflecting phospholipid peroxidation.

### 2.6. ATP Detection

Intracellular ATP levels were measured using an ATP Assay Kit (Beyotime Biotechnology, Cat# S0026) according to the manufacturer’s instructions. Briefly, cells cultured in a 6-well plate were lysed with 200 μL of ATP detection lysis buffer per well. The lysates were centrifuged at 12,000× *g* for 5 min at 4 °C, and the resulting supernatants were collected for analysis. Subsequently, 100 μL of ATP detection working solution was aliquoted into a 96-well white plate and incubated at room temperature for 3–5 min to minimize background signal. Then, 20 μL of each supernatant was added to the wells, mixed rapidly, and the Relative Light Units (RLUs) were measured immediately after a 2-s interval using a luminometer.

### 2.7. Immunofluorescence Staining

Cytoslice cells were rinsed three times with PBS, fixed with 4% paraformaldehyde (PFA) for 1 h, and permeabilized with 0.5% Triton X-100 for 20 min. Primary antibodies against insulin (1:1000, Cell Signaling Technology, 3014, Danvers, MA, USA), the mitochondrial marker Tomm20 (1:2000, Proteintech, 11802-1-AP), and chromogranin A (CHGA, 1:1000, Cell Signaling Technology, 85798) were applied overnight at 4 °C. After washing, cells were incubated with fluorescent secondary antibodies in the dark, counterstained with DAPI, mounted with antifade reagent, and imaged using confocal microscopy.

### 2.8. Annexia V/PI Staining

Cell apoptosis and cell death were assessed using the Annexin V/PI staining kit (Beyotime, ST512) following the manufacturer’s instructions. Islets treated with 100 nm S89 for 48 h were stained with PI working solution for 30 min at room temperature in the dark. Cell death was observed and imaged under a fluorescence microscope.

### 2.9. Immunohistochemistry (IHC)

Paraffin-embedded kidney tissue blocks were sectioned at 5 μm thickness. Sections were dewaxed, rehydrated, and subjected to antigen retrieval using the Tris-EDTA buffer (pH = 8). After blocking using 5% milk for 30 min, sections were incubated overnight at 4 °C with primary antibodies against CHGA (Cell Signaling Technology, 85798) and HIF-1α (ABclonal technology, A11945, Wuhan, China). The next day, sections were washed and incubated with fluorescent secondary antibodies for 2 h. Sections were then counterstained with DAPI, mounted with the antifade reagent, and imaged under a fluorescence microscope.

### 2.10. Hematoxylin–Eosin (H&E) Staining

Kidney paraffin sections (5 μM) were dewaxed, rehydrated, stained with hematoxylin and eosin, dehydrated through ethanol gradients, mounted with neutral resin, and examined via light microscopy to assess histological changes in diabetic or transplanted kidneys.

### 2.11. Mouse Islet Isolation

Mice were euthanized via cervical dislocation, and pancreata were isolated under sterile conditions. Following an abdominal incision, the common bile duct was cannulated, and collagenase P solution was injected to distend the pancreas. Digested tissues were incubated at 37 °C for 10–12 min, quenched with ice-cold RPMI 1640 + 10% FBS, and vigorously shaken. After centrifugation (1000 rpm, 2 min), cell pellets were resuspended in a 1077 density gradient medium, overlaid with serum-free RPMI 1640, and centrifuged at 2400 rpm for 1 min (acceleration 1, deceleration 0). Islet-enriched layers were collected, washed, and hand-picked under microscopy using a 10 μL pipette tip.

### 2.12. Islet Transplantation

Mice were anesthetized with tribromoethanol. After shaving the left abdominal area, islets or cell suspensions were concentrated into small pellets and loaded into PE50 tubes. The tips of the PE50 tubes were inserted under the kidney capsule, and the islets or cell suspensions were slowly injected to ensure even distribution. The peritoneum and skin were sutured, and transplanted mice were regularly monitored for blood glucose levels.

### 2.13. Real-Time Quantitative PCR

Total RNA was extracted using the TRIzol™ reagent (Invitrogen, 15596026, Carlsbad, CA, USA), and RNA concentration was measured spectrophotometrically at 260/280 nm. mRNA was reverse-transcribed into cDNA using a reverse transcription kit (YEASEN, 11151ES60, Shanghai, China) following the manufacturer’s instructions. mRNA expression levels were quantified by real-time PCR. The relative expression levels of target genes were normalized to the endogenous reference gene β-Actin using the 2^−ΔΔCt^ method. Primer sequences are listed in Supplementary Table S1.

### 2.14. Western Blot

Total protein was extracted using the RIPA lysis buffer containing protease inhibitors, incubated on ice with intermittent vortexing, and centrifuged at 12,000× *g* for 10 min at 4 °C. The protein concentration was determined spectrophotometrically, followed by denaturation, SDS-PAGE separation, and transfer to nitrocellulose membranes. Membranes were blocked with 5% skim milk/BSA, then probed overnight at 4 °C with antibodies against BCL-2 (1:500, BD Biosciences, 610539, San Jose, CA, USA), BAX (1:500, BD Biosciences, 610982), Insulin (1:1000, Cell Signaling Technology, 3014), PAX6 (1:1000, ABclonal technology, A7334), and MAFA (1:1000, ABclonal technology, A18662). After washing, HRP-conjugated secondary antibodies were applied for 2 h, and proteins were visualized using the ECL kit. Band intensities were analyzed by ImageJ (1.6.0_20), normalized to β-actin, and repeated three times.

### 2.15. Statistical Analysis

Data are presented as mean values ± SEM. The results were analyzed using ANOVA with GraphPad Prism 8.0.1 (La Jolla, CA, USA). *p* < 0.05 was considered statistically significant.

## 3. Results

### 3.1. S89 Enhanced Mitochondrial Fusion and Cell Viability in Min6 Cells

**Figure 1:**
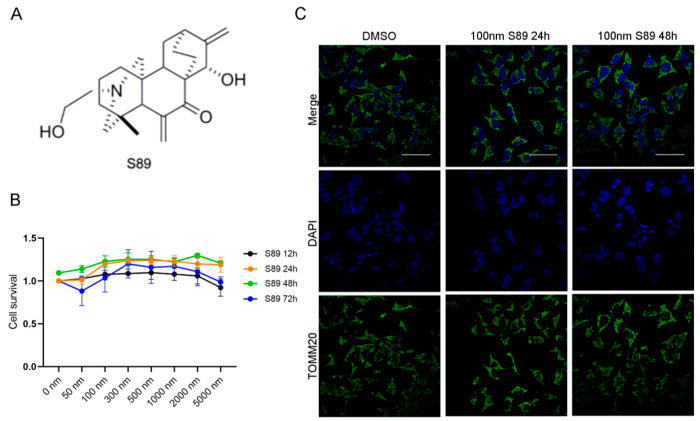
S89 restored cell viability in hypoxia stress-treated Min6 cells. (**A**) Chemical structure of S89. (**B**) The viability of Min6 cells treated with S89 was measured using the CCK-8 assay at different concentrations of S89. Various concentrations of S89, ranging from 50 nM to 5 μM, were added to the culture medium for specified durations before conducting the CCK-8 assay. Cell viability was normalized to that of cells not exposed to S89 at 12 h. (**C**) Microscopic images of mitochondria under S89 treatment. Mitochondrial morphology was assessed using immunofluorescence staining of TOMM20, as shown in green. Cells were counterstained with DAPI, as shown in blue (scale bar = 50 μm).

S89 is a small-molecule compound that promotes mitochondrial fusion, offering a therapeutic strategy for diseases involving mitochondrial fusion defects. Therefore, we sought to investigate whether S89 could protect transplanted islets against hypoxic stress during transplantation. (Figure 1A). To assess the cytoprotective effects of S89 on pancreatic islet β-cells, we first performed the CCK-8 assay to evaluate the impact of various S89 concentrations on Min6 cell viability. The results showed that increasing concentrations of S89 (0, 5, 100, 300, 500, 1000, 2000, and 5000 nM) had no toxic effects on Min6 cells. Instead, cell viability exhibited a dose-dependent enhancement. Based on the CCK-8 assay results, 100 nM S89 treatment for 48 h was selected for subsequent experiments. (Figure 1B). To determine whether S89 induces mitochondrial fusion in Min6 cells consistent with previous reports, we performed immunofluorescence staining for TOMM20, a mitochondrial outer membrane protein. Imaging analysis revealed that S89 treatment significantly increased mitochondrial fusion compared to untreated controls. (Figure 1C). To validate the specificity of S89 as an MFN1 agonist, we assessed its effect on mitochondrial fusion in Min6 cells with different genetic backgrounds. As expected, S89 treatment significantly promoted mitochondrial fusion in both wild-type (WT) and Mfn2 knockdown (KD) Min6 cells. In contrast, S89 failed to induce mitochondrial fusion in *Mfn1* KD Min6 cells. These results demonstrate that the pro-fusion activity of S89 is strictly dependent on MFN1 but not MFN2 (Supplementary Figure S1A).

### 3.2. S89 Alleviated Hypoxia-Induced Mitochondrial Dysfunction in Min6 Cells

**Figure 2:**
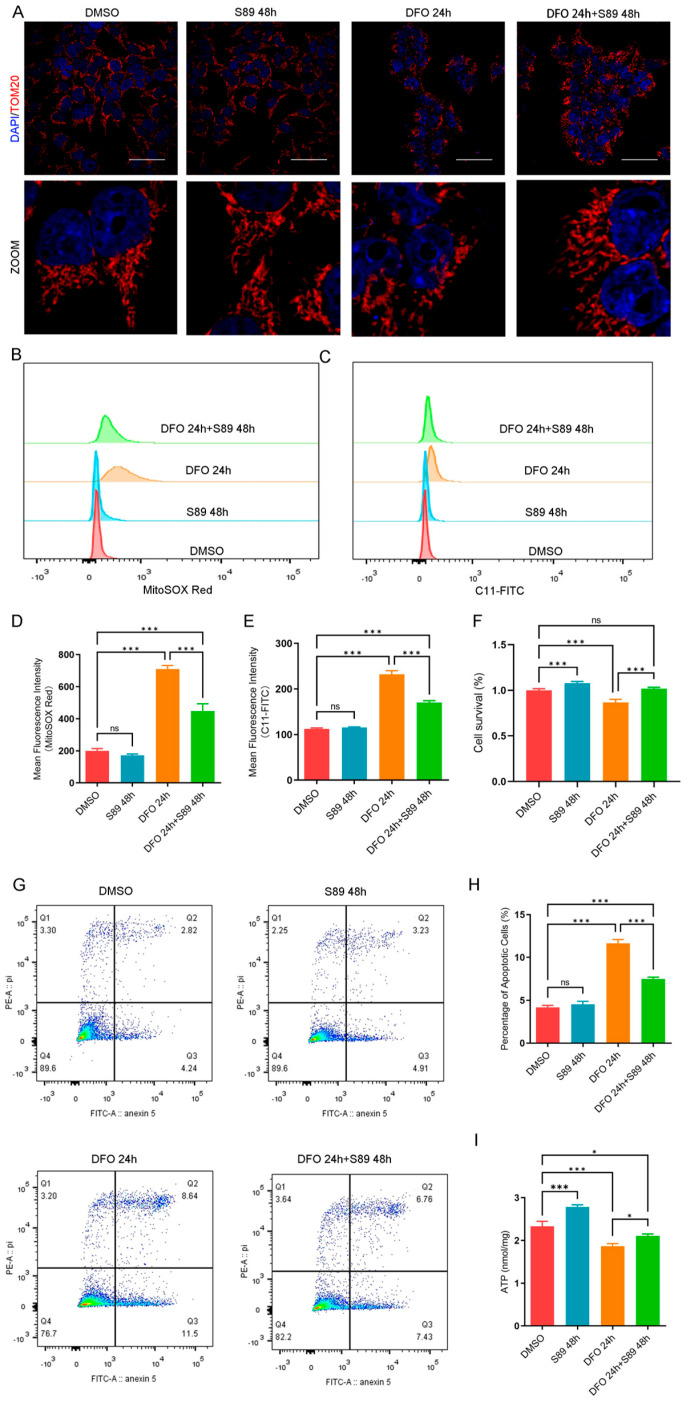
S89 alleviated hypoxia-induced oxidative stress in Min6 cells. (**A**) Microscopic images of mitochondria subjected to S89 treatment under hypoxic stress conditions. Mitochondrial morphology was assessed via immunofluorescence staining of TOMM20; cells were treated with 100 nM deferoxamine (DFO), either alone or in combination with S89. Red, TOMM20. Nuclei were counterstained with DAPI, as shown in blue (scale bar = 50 μm). (**B**–**E**) The levels of mtROS (**B**) and lipid peroxidation (**C**) in Min6 cells were measured through flow cytometry under DFO treatment, both independently and in conjunction with 100 nM S89. (**D**) and (**E**) present the corresponding statistical analyses of (**B**) and (**C**), respectively. Data are presented as the mean ± SEM (n = 3 independent experiments). Statistical significance was determined via one-way ANOVA. (**F**) The cell viability of Min6 cells after supplementation of 100 nM S89 under DFO treatment. Data are presented as the mean ± SEM (n = 3 independent experiments). Statistical significance was determined via one-way ANOVA. (**G**) The Annexin V level in Min6 cells under DFO treatment was detected by flow cytometry, both alone and in combination with 100 nM S89. (**H**) shows the proportional statistics of the apoptosis partition in (**G**). Data are presented as the mean ± SEM (n = 3 independent experiments). Statistical significance was determined via one-way ANOVA. (**I**) The ATP content in Min6 cells was measured after treatment with DFO, both alone and in combination with 100 nM S89. Data are presented as the mean ± SEM (n = 3 independent experiments). Statistical significance was determined via one-way ANOVA. *, *p* < 0.05; ***, *p* < 0.001; ns, *p* > 0.05.

Hypoxia-induced mitochondrial dysfunction and cell death represent critical challenges in islet transplantation. To investigate whether S89 could mitigate hypoxia-induced mitochondrial dysfunction, we first established a chemical hypoxia model using deferoxamine (DFO) treatment in Min6 cells, which mimics hypoxic stress by stabilizing hypoxia-inducible factor-1α (HIF-1α) and disrupting mitochondrial respiration [19]. This model enabled us to evaluate whether S89 alleviates hypoxia-induced perturbations in mitochondrial dynamics. Immunofluorescence data revealed that cells subjected to 24-h hypoxia treatment exhibited significantly increased mitochondrial fragmentation compared to the control group. However, S89 supplementation substantially reduced this hypoxia-induced mitochondrial fragmentation (Figure 2A). To further assess the impact of S89 on hypoxia-induced oxidative stress in Min6 cells, we evaluated mitochondrial reactive oxygen species (mtROS) and lipid peroxidation levels. As shown in Figure 2B–E, hypoxic stress caused significant increases in mtROS and lipid peroxidation, which were significantly reduced by S89 pretreatment. In addition, S89 pretreatment mitigated the hypoxia-induced decline in the viability of Min6 cells (Figure 2F). We also utilized Annexin V and PI staining to determine the effect of S89 on hypoxia-induced apoptosis in Min6 cells. The results indicated that S89 can suppress early DFO-induced apoptosis in Min6 cells (Figure 2G,H). Furthermore, metabolic assays indicate that S89 enhances bioenergetic resilience in Min6 cells by increasing ATP generation under normal conditions and preventing its decline during hypoxic stress, underscoring its efficacy for maintaining energy homeostasis in the context of metabolic challenges (Figure 2I). To validate our findings in a physiologically relevant model, we employed a physical hypoxia system (1% O_2_). Consistent with the results from the DFO model, the S89 treatment effectively attenuated mitochondrial fragmentation, suppressed the accumulation of mtROS and lipid peroxidation, and restored ATP levels under hypoxic conditions (Supplementary Figure S2). Notably, exposure to 1% O_2_ for 48 h did not induce significant cell death in Min6 cells, suggesting the intrinsic resistance of this cell line to acute physical hypoxia. These data collectively demonstrate the protective efficacy of S89 against mitochondrial dysfunction in both chemical and physical hypoxia models.

### 3.3. S89 Inhibited Hypoxia Stress-Induced Apoptosis in Islets

**Figure 3:**
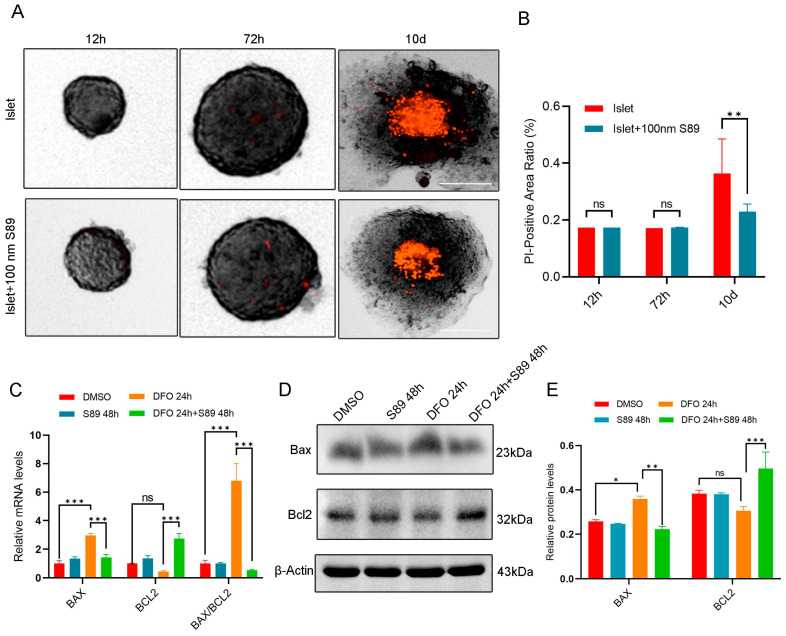
S89 inhibited Min6 cell apoptosis induced by hypoxic stress. (**A**) Microscopic images showing morphology and cell death of pancreatic islet organoids supplemented with 100 nM S89. After isolating mouse primary pancreatic islet cells, pancreatic islet cell death levels were detected using propidium iodide (PI) at 12 h, 72 h, and 10 d. This is represented in red, PI (scale bar = 50 μm). (**B**) Statistical analysis of the death level of mouse primary pancreatic islet cells. Data are presented as the mean ± SEM (n = 5 independent experiments). Statistical significance was determined via one-way ANOVA. (**C**) The mRNA levels of BAX and BCL-2 were determined using RT-qPCR after supplementation of S89 with 100 nM DFO treatment. Data are presented as the mean ± SEM (n = 3 independent experiments). Statistical significance was determined via one-way ANOVA. (**D**) The protein levels of BAX and BCL-2 were examined using Western blotting after supplementation of S89 under 100 nM DFO treatment. (**E**) Statistical analysis of the gray values in Western Blot results. Data are presented as the mean ± SEM (n = 3 independent experiments). Statistical significance was determined via one-way ANOVA. *, *p* < 0.05; **, *p *< 0.01; ***, *p* < 0.001; ns, *p* > 0.05.

To assess hypoxia-induced cell death, we performed propidium iodide (PI) staining to detect the loss of membrane integrity in islet cells. Compared to normoxic controls, hypoxia-stressed cells exhibited a significant increase in PI-positive cells. S89 supplementation substantially attenuated this hypoxia-induced rise in PI-positive cell counts (Figure 3A,B). To assess the regulation of mitochondrial apoptosis during hypoxia, we measured the expression levels of pro-apoptotic BAX and anti-apoptotic BCL-2 proteins. RT-quantitative PCR and Western Blot revealed that hypoxia stress (induced by DFO treatment) increased BAX expression while decreasing BCL-2 expression in Min6 cells; by contrast, co-treatment with S89 significantly attenuated these alterations by elevating BCL-2 levels and reducing BAX expression compared to DFO treatment alone (Figure 3C,D). These findings indicate S89’s protective effect against hypoxia stress-induced apoptosis in Min6 cells.

### 3.4. S89 Promoted Insulin Secretion in Pancreatic Islet Cells

**Figure 4:**
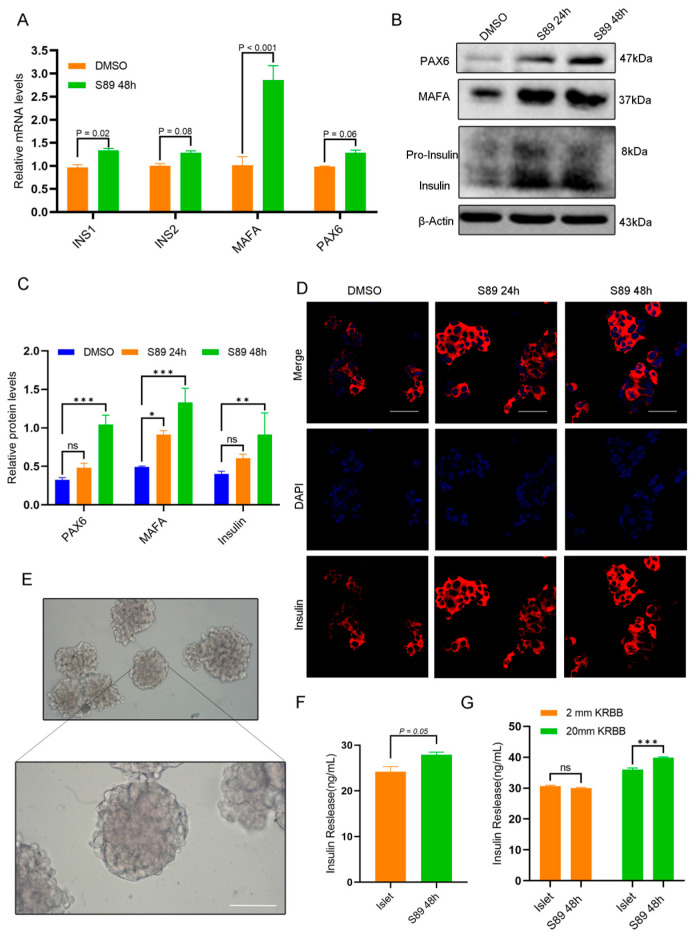
S89 promotes insulin secretion in Min6 and mouse primary pancreatic islet cells. (**A**) The mRNA levels of insulin secretion-related genes (INS1, INS2, MAFA, PAX6) were determined using RT-qPCR after supplementation of 100 nM S89. Data are presented as the mean ± SEM (n = 3 independent experiments). Statistical significance was determined via one-way ANOVA. (**B**) The protein levels of genes related to insulin secretion (Insulin, MAFA, PAX6) were determined using Western blotting after supplementation of 100 nM S89. (**C**) Statistical analysis of the gray values in Western Blot results. Data are presented as the mean ± SEM (n = 3 independent experiments). Statistical significance was determined via one-way ANOVA. (**D**) The insulin level was determined using immunofluorescence staining after supplementing 100 nM S89. Insulin is shown in red (scale bar = 50 μm). (**E**) Representative image of mouse primary pancreatic islet cells (scale bar = 100 μm). (**F**) Insulin levels were measured in the supernatant of the culture medium 48 h after supplementing 100 nM S89. Data are presented as the mean ± SEM (n = 3 independent experiments). Statistical significance was determined via one-way ANOVA. (**G**) Mouse primary pancreatic islet cells were stimulated with low-concentration (2 mM) and high-concentration (20 mM) glucose to detect insulin levels in the culture medium. Data are presented as the mean ± SEM (n = 3 independent experiments). Statistical significance was determined via one-way ANOVA. *, *p* < 0.05; **, *p* < 0.01; ***, *p* < 0.001; ns, *p* > 0.05.

Mitochondria play a critical role in regulating energy metabolism and mediating cell signaling. Mitochondrial dysfunction can reduce cellular energy production, impair insulin signaling, decrease tissue insulin sensitivity, and disrupt glucose homeostasis. To evaluate S89’s impact on insulin biosynthesis in Min6 cells, we quantified the expression of insulin-related genes using RT-qPCR and Western Blot [20,21,22,23]. RT-qPCR data showed that S89 treatment upregulated the expression of INS1, INS2, MAFA, and PAX6 mRNA (Figure 4A). Western Blot analysis also confirmed increased protein expression of INS, MAFA, and PAX6 (Figure 4B). To further explore S89 functional effects in Min6 cells, we assessed insulin gene expression via immunofluorescence. As shown in Figure 4C, compared to the control group, S89 treatment significantly enhanced insulin expression in Min6 cells. Additionally, we isolated mouse islet cells to confirm S89’s functional effects (Figure 4D). ELISA quantification revealed elevated insulin levels in supernatants after 48-h treatment with 100 nM S89 isolated in primary mouse islets (Figure 4E). Additionally, we evaluated β-cell function in S89-treated islets using glucose-stimulated insulin secretion (GSIS) assays. The results suggest that S89 treatment promoted insulin secretion specifically under high glucose (20 mM) conditions, while showing no clear effect at low glucose levels (2 mM) (Figure 4F). This glucose-dependent potentiation appears to be mediated through S89-induced mitochondrial fusion, which directly increases ATP production (Figure 2I), thus contributing to enhanced second-phase insulin release under sustained high-glucose stimulation.

### 3.5. S89 Maintains Blood Glucose Homeostasis in T1D Mice

**Figure 5:**
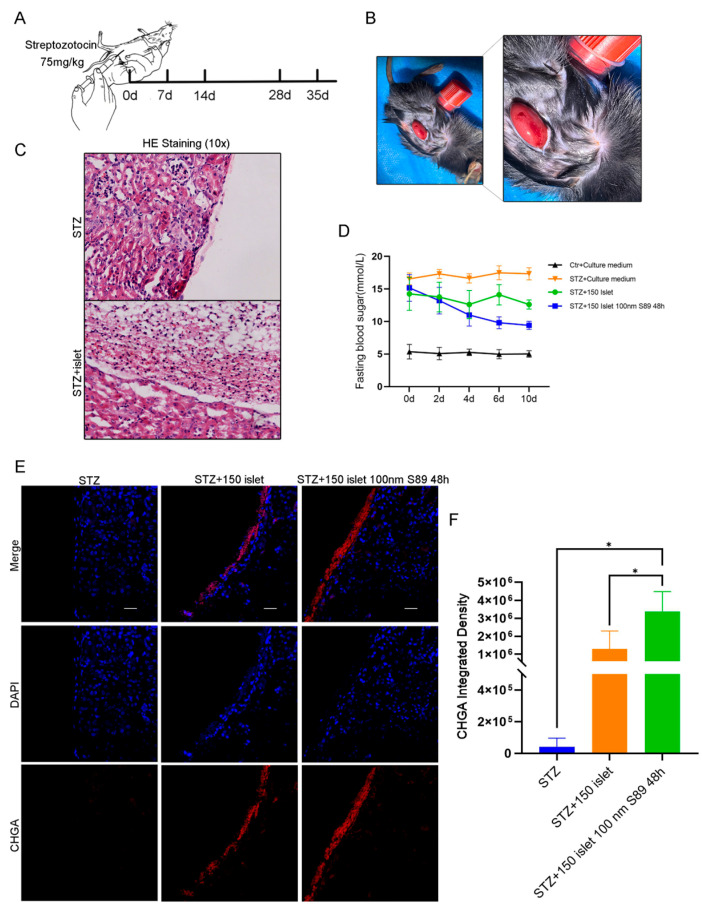
S89 pretreatment of pancreatic islets maintains blood glucose homeostasis in T1D mice. (**A**) Construction of the T1D mouse model. (**B**) Representative images of mouse renal capsule transplantation of pancreatic islets. (**C**) After the transplantation of pancreatic islets in T1D mice, the morphology of renal capsule tissue was detected by hematoxylin and eosin staining. (**D**) After transplanting pancreatic islets in T1D mice for 0 d, 2 d, 4 d, 6 d, and 10 d, fasting blood glucose levels were measured by tail vein blood collection, n = 4. (**E**) After transplantation of pancreatic islets in T1D mice, immunofluorescence staining was used to detect endocrine cells in renal paraffin sections. DAPI is shown in blue and CHGA is shown in red (scale bar = 10 μm). (**F**) Statistical analysis of red fluorescence in mouse kidney grafts. Data are presented as the mean ± SEM (n = 3 independent experiments). Statistical significance was determined via one-way ANOVA. *, *p* < 0.05.

Building on in vitro evidence that S89 enhances hypoxia resistance in Min6 cells and augments insulin biosynthesis and secretion in mouse islets, we investigated whether S89 preconditioning improves islet transplantation outcomes. To test this, we established a streptozotocin (STZ)-induced T1D mouse model (Figure 5A) [24]. Primary mouse islets were pretreated with 100 nm S89 for 48 h or left untreated and then transplanted under the renal capsule of diabetic mice to compare the therapeutic efficacy of S89-preconditioned grafts with that of untreated controls (Figure 5B,C). To sensitively assess the impact of S89 on islet graft function, a sub-therapeutic marginal mass of 150 islets (below the typical therapeutic threshold) was transplanted. This design limits the intrinsic glycemic-normalizing capacity of the islets, thereby enhancing the detectability of improvements conferred by S89 co-treatment. Control mice that received untreated islets showed a modest reduction in blood glucose levels post-transplantation. By contrast, mice transplanted with S89-pretreated islets exhibited significantly lower blood glucose levels, with the most pronounced reduction observed on day 10 (*p* < 0.05 vs. controls) (Figure 5D). Immunofluorescence staining for chromogranin A (a marker of endocrine cell mass) and insulin also revealed thicker and more structurally preserved islet clusters in the renal subcapsule of S89-treated mice. This mitigated their hypoxic condition, indicating enhanced graft survival (Figure 5E and Supplementary Figure S1B,C). These results demonstrate that S89 pretreatment enhances islet graft functionality and contributes to the stabilization of glucose homeostasis in T1D mice.

## 4. Discussion

Islet transplantation represents a promising therapeutic strategy for insulin-deficient diabetes, enabling the restoration of physiological insulin secretion [25]. However, hypoxic stress triggers cellular stress responses, compromises islet viability, and ultimately diminishes engraftment efficacy after the transplant [26]. Crucially, hypoxia-induced oxidative stress directly impairs mitochondrial function, as evidenced by collapsed membrane potential, disrupted oxidative phosphorylation, and aberrant fission–fusion dynamics [27,28]. This mitochondrial dysfunction, in turn, compromises islet cell viability and insulin secretion capacity, representing a persistent challenge in transplantation biology [29]. Mitochondria are dynamic organelles that continuously undergo fusion and fission to maintain cellular homeostasis. Fusion preserves organellar integrity by redistributing mtDNA, proteins, and metabolites across the mitochondrial network, diluting localized damage, and restoring bioenergetic capacity [30]. Conversely, fission segregates dysfunctional segments via asymmetric division, generating depolarized fragments that are degraded through mitophagy to prevent propagation of dysfunctions [31]. Therefore, therapeutic strategies that enhance mitochondrial fusion can restore mitochondrial function and cellular homeostasis. These strategies demonstrate significant potential for treating diverse pathologies linked to mitochondrial dysfunction. Although both MFN1 and MFN2 are key proteins mediating mitochondrial outer membrane fusion, their functions are not redundant [32]. Growing evidence suggests that MFN1 is the primary driver of GTPase activity and fusion dynamics, while MFN2 plays unique roles in mitochondrial–ER tethering and the regulation of mitophagy, among other functions [33]. Therefore, targeting the S89 site of MFN1 may directly and efficiently promote the physical fusion of the mitochondrial network, resulting in rapid improvements for mitochondrial energy metabolism and quality control. By contrast, MFN2 agonists may have a more significant role in mitochondrial–ER coupling-related cellular signaling. The fission inhibitor Mdivi-1 prevents mitochondrial division by inhibiting the GTPase DRP1; however, a potential limitation of this strategy is that it may impede necessary healthy fission processes (such as the clearance of damaged mitochondria via asymmetric fission-induced mitophagy and mitochondrial biogenesis) [34]. As a small-molecule agonist that specifically activates MFN1, S89 mitigates mitochondrial stress in disease models [17]. To evaluate its role in islet cells, we assessed S89 in Min6 cells, observing no cytotoxicity or concentration-dependent enhancement of cell viability. Given S89’s pro-fusion activity, we also investigated its capacity to prevent hypoxia-induced mitochondrial fragmentation. We found that S89 treatment attenuated mitochondrial fragmentation while concurrently reducing ROS overaccumulation and lipid peroxidation, indicating the existence of potent antioxidant protection against hypoxia-induced damage in Min6 cells.

Pancreatic β-cells exhibit intrinsic vulnerability to oxidative stress due to their low expression of key antioxidant enzymes (e.g., superoxide dismutase). This makes them highly susceptible to mitochondrial reactive oxygen species (mtROS)-induced apoptosis [35]. Within this context, mitochondrial dynamics critically influence cell fate: fragmentation sensitizes mitochondria to pro-apoptotic signals such as BAX insertion, while fusion confers resistance by diluting localized damage [36,37,38]. Small-molecule S89 addresses this by enhancing mitochondrial fusion through the activation of mitofusin-1 (MFN1). The stabilization of the mitochondrial network improves electron transport chain efficiency and reduces the generation of mtROS at its source, thereby shifting the BCL-2/BAX equilibrium toward cell survival. Specifically, our immunohistochemical analysis showed that S89 treatment significantly lowered HIF-1α levels in transplanted islets, suggesting that the reduction in mtROS prevents its pathological stabilization. These findings indicate that the mtROS–HIF-1α axis is a crucial downstream pathway through which S89-mediated mitochondrial fusion enhances β-cell survival during metabolic stress.

S89 significantly enhances islet β-cell function by upregulating insulin biosynthesis genes in Min6 cells and improving glucose-stimulated insulin secretion (GSIS) in primary murine islets. Transplantation of S89-pretreated islets into type-1 diabetic (T1D) mice accelerated the restoration of normoglycemia within 10 days, demonstrating superior glycemic control. While established mitochondrial protection strategies in islet transplantation, such as the use of oxygenated perfluorocarbons for oxygen delivery or N-acetylcysteine (NAC) as a direct antioxidant, primarily aim to mitigate the consequences of ischemic and oxidative stress, S89 represents a mechanistically distinct approach. NAC, a precursor of glutathione, functions as a direct reactive oxygen species (ROS) scavenger that transiently neutralizes free radicals and replenishes the cellular antioxidant pool. In contrast, S89 targets the root cause of oxidative damage by activating MFN1, which promotes mitochondrial fusion and thereby restores structural integrity. The innate resilience of the organelle is enhanced, improving electron transport chain efficiency and fundamentally reducing ROS generation at its source. As such, S89 moves beyond symptomatic relief toward fostering endogenous mitochondrial stability, offering a potentially more sustainable strategy for enhancing islet graft survival.

## 5. Conclusions

This study demonstrates that the small-molecule MFN1 agonist S89 effectively protects pancreatic β-cells against hypoxia-induced damage by promoting mitochondrial fusion. This intervention mitigates the overaccumulation of mitochondrial reactive oxygen species and lipid peroxidation, thereby attenuating the mitochondrial apoptotic pathway and enhancing cell survival. Notably, S89 pretreatment not only enhanced the hypoxic resistance of islets in vitro but also significantly improved their engraftment and function in a murine model of type 1 diabetes. This led to improved restoration of glycemic homeostasis. However, further experiments are required to validate aspects such as in vivo toxicity and applicability to human islets. Specifically, while our current study in diabetic mouse models demonstrates a promising safety profile for S89, its long-term systemic toxicity warrants further investigation in chronic administration models. Additionally, dose–response studies on primary human islets are essential to confirm their efficacy and establish an optimal therapeutic window for clinical translation. Finally, comprehensive off-target profiling should be conducted to ensure the specificity of S89 as an MFN1 agonist and to rule out any unintended interactions. In summary, S89 represents a “foundational repair” strategy, enhancing endogenous oxidative buffering capacity. It is positioned as a promising therapeutic candidate for mitigating ischemia-related islet damage and improving transplant outcomes.

## Data Availability

All data generated or analyzed during this study are included in the published article and its Supplementary Information files.

## Supplementary Materials

The following supporting information can be downloaded at https://www.mdpi.com/article/10.3390/biom15111585/s1. Figure S1: (A) Microscopic images of mitochondria under S89 treatment. Mitochondrial morphology was assessed using immunofluorescence staining of TOMM20, as shown in green. Cells were counterstained with DAPI, as shown in blue (scale bar = 100 px). (B) After transplantation of pancreatic islets in T1D mice, immunofluorescence staining was used to detect endocrine cells in renal paraffin sections. DAPI is shown in blue and insulin is shown in red (scale bar = 100 μm). (C) Statistical analysis of red fluorescence in mouse kidney grafts. Data are presented as the mean ± SEM (n = 3 independent experiments). Statistical significance was determined via one-way ANOVA. (D) After transplanting islets into type 1 diabetic mice, the HIF-1α content in the renal capsular tissue was detected via immunohistochemistry; Figure S2: S89 alleviated hypoxia-induced oxidative stress in Min6 cells; Table S1. The primer sequences.

## Abbreviations

The following abbreviations are used in this manuscript:

MFN1	Mitofusin 1
STZ	Streptozotocin
T1D	Type 1 diabetic
PFA	Paraformaldehyde
DFO	Deferoxamine
mtROS	Mitochondrial reactive oxygen species
HIF-1α	Hypoxia-inducible factor-1α
PI	Propidium iodide
GSIS	Glucose-stimulated insulin secretion
IHC	Immunohistochemistry
Min6	Mouse insulinoma cells
DMSO	Dimethyl sulfoxide
