# Supplementary material for: A Small-Molecule Mitofusin 1 Agonist Enhances Islet Survival Under Hypoxic Conditions In Vitro and Improves Transplantation Outcomes
Source: Biomolecules. 2025 Nov 11;15(11):1585. doi: 10.3390/biom15111585 (PMC12649836; doi:10.3390/biom15111585)
Supplement: Supplementary file 1 [file biomolecules-15-01585-s001.zip › biomolecules-3878693-supplementary.pdf]

Table S1. The primer sequences.

Gene	Primer Sequences (5’-3’)
β-Actin	F: CATTGCTGACAGGATGCAGAAGG<br>R: TGCTGGAAGGTGGACAGTGAGG
INS1	F: CGTGGCTTCTTCTACACACCCA<br>R: TGCAGCACTGATCCACAATGCC
MAFA	F: GCTTCAGCAAGGAGGAGGTCAT<br>R: TCTCGCTCTCCAGAATGTGCCG
PAX6	F: CTGAGGAACCAGAGAAGACAGG<br>R: CATGGAACCTGATGTGAAGGAGG
INS2	F: CGTGGCTTCTTCTACACACCCA<br>R: TCCAGTGCCAAGGTCTGAAGGT
BAX	F: AGGATGCGTCCACCAAGAAGCT<br>R: TCCGTGTCCACGTCAGCAATCA
BCL2	F: CCTGTGGATGACTGAGTACCTG<br>R: AGCCAGGAGAAATCAAACAGAGG

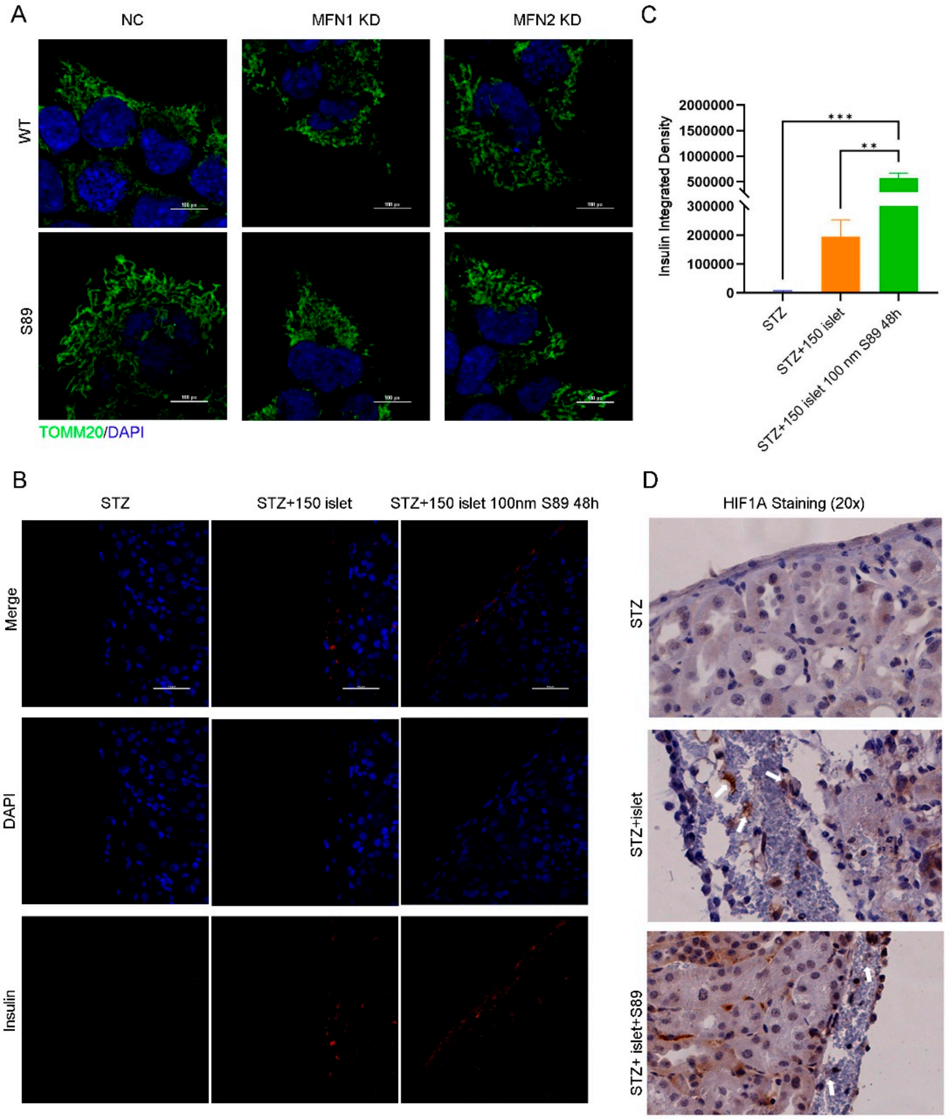

Supplementary Figure S1. (**A**) Microscopic images of mitochondria under S89 treatment. Mitochondrial morphology was assessed using immunofluorescence staining of TOMM20, as shown in green. Cells were counterstained with DAPI, as shown in blue (scale bar = 100 px). (**B**) After transplantation of pancreatic islets in T1D mice, immunofluorescence staining was used to detect endocrine cells in renal paraffin sections. DAPI is shown in blue and insulin is shown in red (scale bar = 100 μm). (**C**) Statistical analysis of red fluorescence in mouse kidney grafts. Data are presented as the mean ± SEM (n = 3 independent experiments). Statistical significance was determined via one-way ANOVA. (**D**) After transplanting islets into type 1 diabetic mice, the HIF-1α content in the renal capsular tissue was detected via immunohistochemistry.

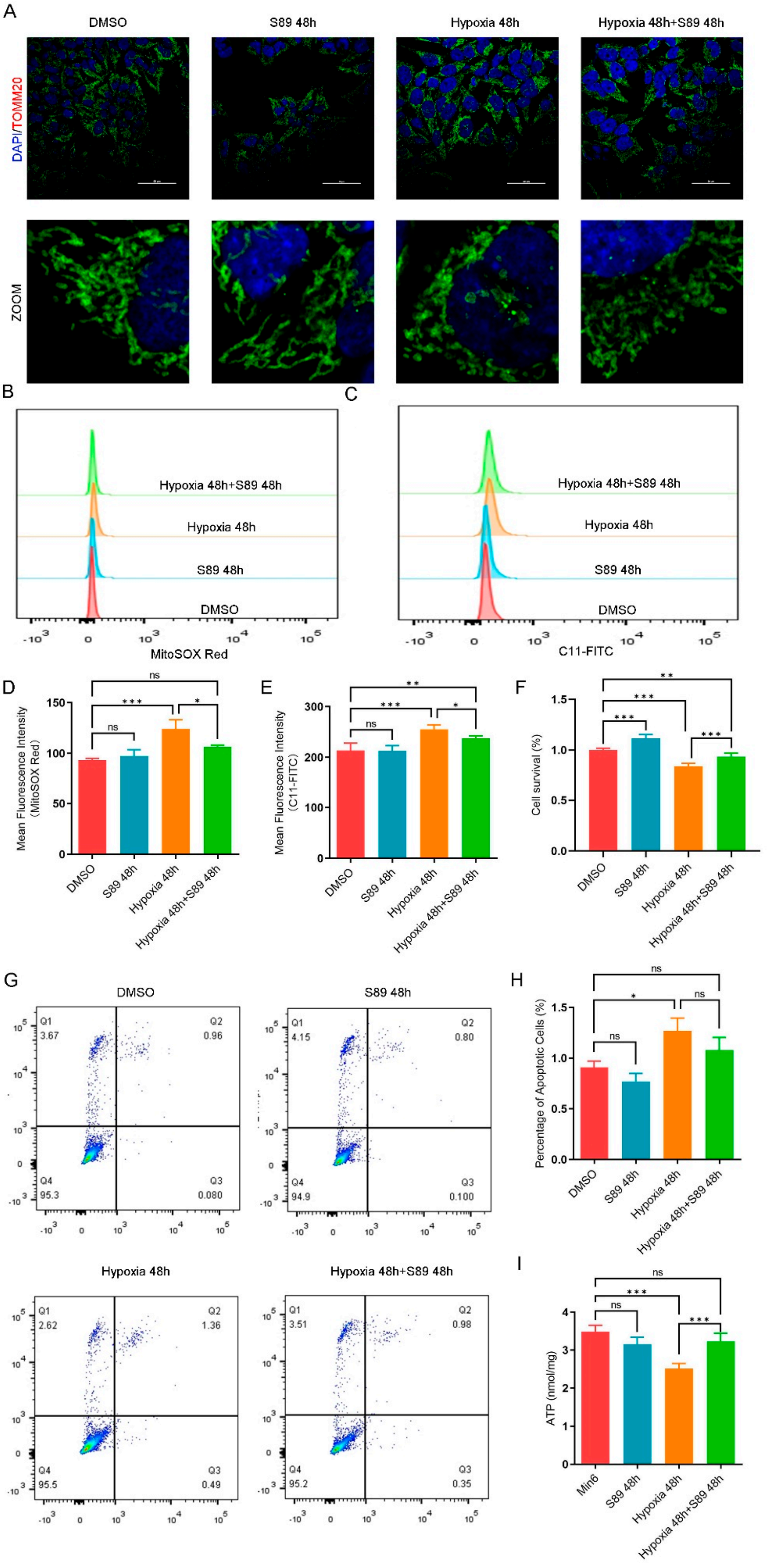

Supplementary Figure S2. S89 alleviated hypoxia-induced oxidative stress in Min6 cells. (**A**) Microscopic images of mitochondria subjected to S89 treatment under hypoxic stress conditions. Mitochondrial

morphology was assessed via immunofluorescence staining of TOMM20, with cells exposed to 1% O_2 for 48 h, either alone or in combination with S89 (represented in red). Nuclei were counterstained with DAPI, as shown in blue (scale bar = 50 μm). (**B**–**E**) The levels of mtROS (B) and lipid peroxidation (**C**) in Min6 cells were measured through flow cytometry under 1% O_2 for 48 h, both independently and in conjunction with 100 nM S89. (**D**) and (**E**) present the corresponding statistical analyses of (**B**) and (**C**), respectively. Data are presented as the mean ± SEM (n = 3 independent experiments). Statistical significance was determined via one-way ANOVA. (**F**) The cell viability of Min6 cells after supplementation of 100 nM S89 under 1% O_2 for 48 h. Data are presented as the mean ± SEM (n = 3 independent experiments). Statistical significance was determined via one-way ANOVA. (**G**) The Annexin V level in Min6 cells was detected by flow cytometry under 1% O_2 for 48 h, both alone and in combination with 100 nM S89. (**H**) shows the proportional statistics of the apoptosis partition in (**G**). Data are presented as the mean ± SEM (n = 3 independent experiments). Statistical significance was determined via one-way ANOVA. (**I**) The ATP content in Min6 cells was measured after treatment with 1% O_2 for 48 h, both alone and in combination with 100 nM S89. Data are presented as the mean ± SEM (n = 3 independent experiments). Statistical significance was determined via one-way ANOVA.
