# Supplementary material for: A Small-Molecule Mitofusin 1 Agonist Enhances Islet Survival Under Hypoxic Conditions In Vitro and Improves Transplantation Outcomes
Source: Biomolecules. 2025 Nov 11;15(11):1585. doi: 10.3390/biom15111585 (PMC12649836; doi:10.3390/biom15111585)

Fig 3D

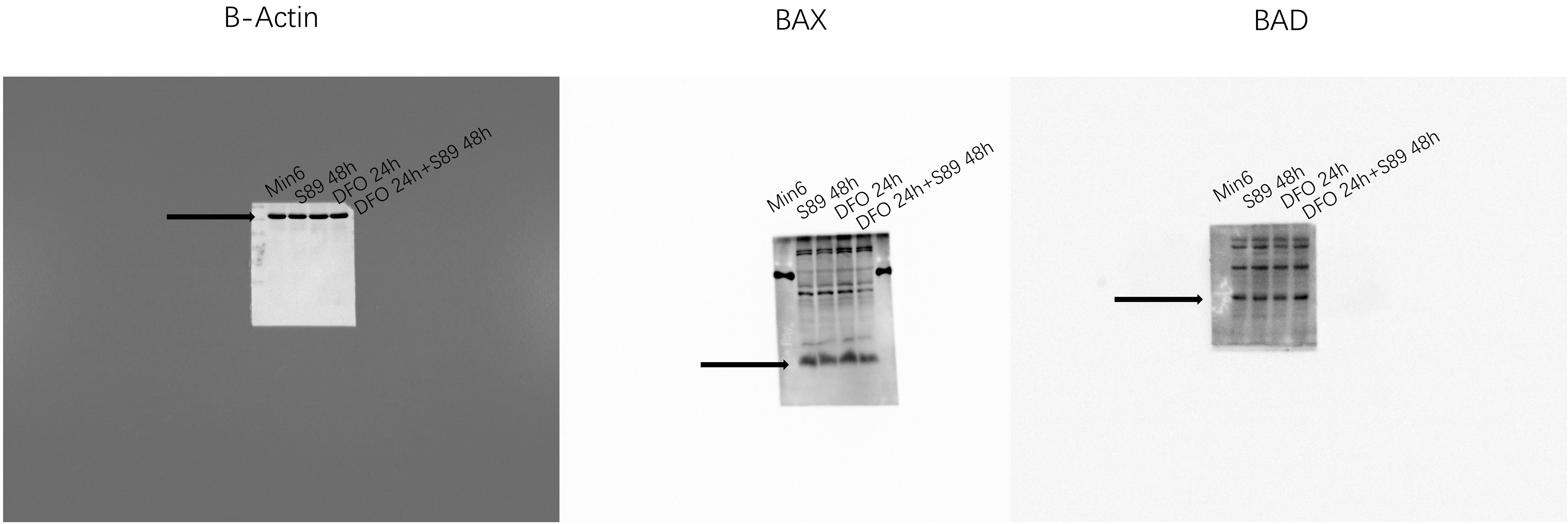
B-Actin
BAX
BAD
Min6
S89 48h
DFO 24h
DFO 24h+S89 48h
Min6
S89 48h
DFO 24h
DFO 24h+S89 48h
Min6
S89 48h
DFO 24h
DFO 24h+S89 48h

Fig 4B

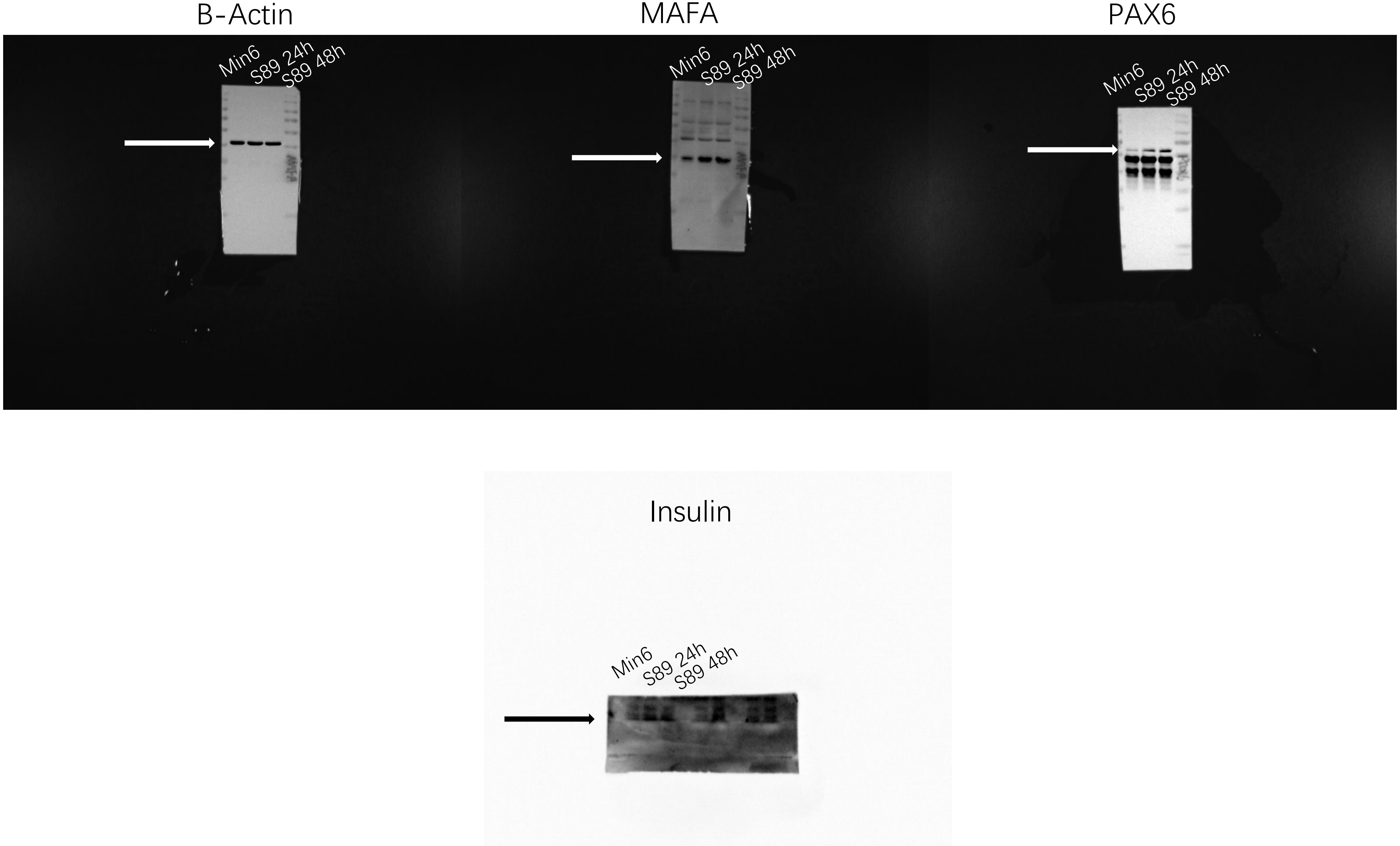

Supplement: Supplementary file 1 [file biomolecules-15-01585-s001.zip › Western Blotting Original Images.zip!/Original Images.pdf]
